# Comprehensive genomic landscape of antibiotic resistance in *Staphylococcus epidermidis*

**DOI:** 10.1128/msystems.00226-24

**Published:** 2024-05-10

**Authors:** Do-Hoon Lee, Kihyun Lee, Yong-Seok Kim, Chang-Jun Cha

**Affiliations:** 1 Department of Systems Biotechnology and Center for Antibiotic Resistome, Chung-Ang University, Anseong, South Korea; University of California San Diego, La Jolla, California, USA

**Keywords:** *Staphylococcus epidermidis*, antibiotic resistance, staphylococci, comparative genomics, virulence factor, *Staphylococcus aureus*

## Abstract

*Staphylococcus epidermidis*, a common commensal bacterium found on human skin, can cause infections in clinical settings, and the presence of antibiotic resistance genes (ARGs) impedes the treatment of *S. epidermidis* infections. However, studies characterizing the ARGs in *S. epidermidis* with regard to genomic and ecological diversities are limited. Thus, we performed a comprehensive and comparative analysis of 405 high-quality *S. epidermidis* genomes, including those of 35 environmental isolates from the Han River, to investigate the genomic diversity of antibiotic resistance in this pathogen. Comparative genomic analysis revealed the prevalence of ARGs in *S. epidermidis* genomes associated with multi-locus sequence types. The genes encoding dihydrofolate reductase (*dfrC*) and multidrug efflux pump (*norA*) were genome-wide core ARGs. β-Lactam class ARGs were also highly prevalent in the *S. epidermidis* genomes, which was consistent with the resistance phenotype observed in river isolates. Furthermore, we identified chloramphenicol acetyltransferase genes (*cat*) in the plasmid-like sequences of the six river isolates, which have not been reported previously in *S. epidermidis* genomes. These genes were identical to those harbored by the *Enterococcus faecium* plasmids and associated with the insertion sequence 6 family transposases, homologous to those found in *Staphylococcus aureu*s plasmids, suggesting the possibility of horizontal gene transfer between these Gram-positive pathogens. Comparison of the ARG and virulence factor profiles between *S. epidermidis* and *S. aureu*s genomes revealed that these two species were clearly distinguished, suggesting genomic demarcation despite ecological overlap. Our findings provide a comprehensive understanding of the genomic diversity of antibiotic resistance in *S. epidermidis*.

**IMPORTANCE:**

A comprehensive understanding of the antibiotic resistance gene (ARG) profiles of the skin commensal bacterium and opportunistic pathogen *Staphylococcus epidermidis* needs to be documented from a genomic point of view. Our study encompasses a comparative analysis of entire *S. epidermidis* genomes from various habitats, including those of 35 environmental isolates from the Han River sequenced in this study. Our results shed light on the distribution and diversity of ARGs within different *S. epidermidis* multi-locus sequence types, providing valuable insights into the ecological and genetic factors associated with antibiotic resistance. A comparison between *S. epidermidis* and *Staphylococcus aureus* revealed marked differences in ARG and virulence factor profiles, despite their overlapping ecological niches.

## INTRODUCTION

Staphylococci are classified based on their ability for blood plasma clotting for clinical diagnosis. *Staphylococcus aureus*, a classical pathogen responsible for a broad range of infections, is a typical coagulase-positive staphylococci (1). It can invade surgical and other wounds, sometimes causing sepsis. Despite its pathogenicity, *S. aureus* is usually harmless on the skin of approximately 30% of healthy individuals and survives well on inanimate surfaces, facilitating further infection (2).

*Staphylococcus epidermidis*, *S. haemolyticus*, and *S. saprophyticus* are well-known coagulase-negative staphylococci (CoNS). With the increase in the incidence of CoNS infection, there is growing awareness that they should be considered pathogens (3). CoNS members are more frequently isolated from patients in the intensive care unit than *S. aureus* during bloodstream infections (4). Historically, they have been considered culture contaminants, as they are widely distributed throughout the human body, including in the mouth and throat. These characteristics render the ability to distinguish between the infecting strains and normal bacterial flora difficult and recognition of their infection challenging. Most symptoms of CoNS infection differ markedly from those of *S. aureus* and are usually subacute or chronic with no apparent symptoms (5). CoNS infections rarely develop into life-threatening cases if treated promptly and appropriately, but in immunocompromised patients, fatal outcomes can occur (6).

*S. epidermidis* is a common human skin commensal bacterium isolated from the entire body surface of healthy individuals (7). Previously, *S. epidermidis* was considered a beneficial skin commensal bacterium involved in promoting homeostasis, defense against pathogens, and skin development (8). Currently, *S. epidermidis* is considered an opportunistic pathogen under certain conditions in which the skin barrier is disrupted by either genetic mutations or physical means (8, 9). The infections usually do not develop into life-threatening diseases due to their low toxicity compared to *S. aureus*; however, they are considered important in the clinical setting because of the high infection frequency and complexity of treatment required (9). Approximately 250,000 intravascular catheter-associated bloodstream infections are reported annually in the USA with *S. epidermidis* being the major cause of sepsis (10).

One of the challenges in treating infections caused by *S. epidermidis* is the presence of antibiotic resistance genes (ARGs). Clinical studies have revealed that antibiotic-resistant *S. epidermidis* strains associated with sepsis and orthopedic infections possess specific genotypes, and they are ubiquitous in clinical settings (11, 12). Notably, *S. epidermidis* strains isolated from patients with orthopedic infections harbor a higher proportion of ARGs and mobile genetic elements (MGEs) than strains isolated from healthy individuals or normal skin that are not the sites of infection (13). Furthermore, multi-locus sequence typing (MLST) analysis revealed the presence of 1,133 sequence types (STs) in *S. epidermidis*, indicating substantial strain-level diversity (14). However, previous studies on *S. epidermidis* have predominantly focused on clinical isolates, such as ST2 and ST23 strains of *S. epidermidis* associated with the nosocomial infection with limited representation of its genetic and ecological diversity. Considering the increasing prevalence of *S. epidermidis* infections in clinical settings, a genome-wide comparative analysis of *S. epidermidis* strains isolated from various sources should be performed to improve our understanding regarding the transmission dynamics of ARGs with the One-Health perspective (15, 16).

Here, we aimed to perform a comparative analysis of ARGs in 405 high-quality *S. epidermidis* genome sequences, including those from 35 isolates from the Han River, South Korea. Our study included both clinical and environmental isolates, enabling a more comprehensive evolutionary assessment of the genomic diversity of antibiotic resistance in *S. epidermidis*. We also compared the ARGs and virulence factors (VFs) present in *S. epidermidis* genomes with those in *S. aureus*, the results of which suggested genomic demarcation despite their ecological overlap.

## RESULTS

### Whole-genome sequences of *S. epidermidis* strains isolated from the Han River

In total, 35 strains of *S. epidermidis* were isolated from the Han River, South Korea. The 16S rRNA gene sequences of the isolates showed the highest similarities to *S. epidermidis* NCTC 11047^T^ (99.1%–100%). The whole-genome sequences were determined using the Oxford Nanopore Technologies sequencing platform. The assembled genomes were generally of high quality with a median of four contigs. The average nucleotide identity (ANI) values of the genomes with *S. epidermidis* NCTC 11047^T^ ranged from 99.5% to 96.8%, indicating that all strains were taxonomically identified to be *S. epidermidis* (17). The genomic features of *S. epidermidis* isolates are summarized in Table S1. The 35 draft genomes were approximately 2.5 ± 0.18 Mbp in size with an average GC content of 32.1%. Each genome contained 2,554 ± 314 genes, consisting of 2,474 ± 314 protein-coding genes, 19 rRNA genes, and 60 ± 1 tRNA genes (Table S1).

### Phylogenomic analyses of *S. epidermidis*

**Fig 1:**
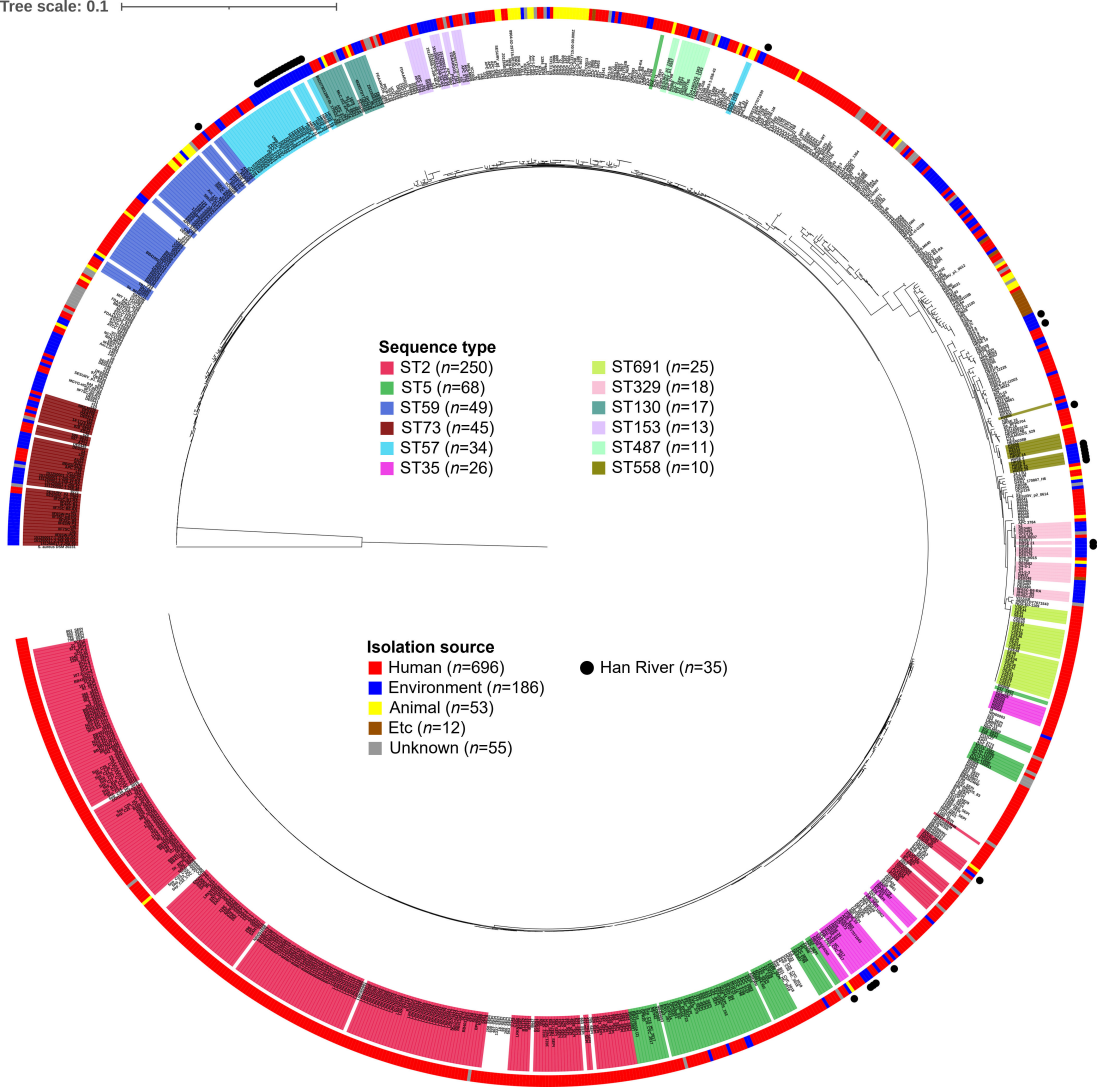
Whole-genome phylogeny of *Staphylococcus epidermidis* associated with their sequence types and isolation sources. A genome-based phylogenetic tree was reconstructed by the maximum-likelihood method using the concatenated alignments of 640 core genes among *S. epidermidis* genomes. *S. aureus* DSM 20231 was used as an outgroup. sequence types with *n* ≥ 10 among the *S. epidermidis* genomes are color-shaded on strain names. Colors in the outer circle indicate the isolation source of each strain. Etc indicates isolation sources other than humans, animals, and the environment. Han River isolates are marked with black circles.

A phylogenomic tree was constructed using the concatenated sequences of 640 core genes shared among the 1,002 *S*. *epidermidis* genomes (Fig. 1). The results showed that genome phylogeny was closely associated with the sequence type. Most *S. epidermidis* strains were isolated from humans, and ST2 was the most abundant lineage, which is known to spread globally in recent years (14). In contrast, hospital-acquired ST23 lineage, which has recently emerged as a significant global threat to patient health, accounted for a very low percentage (0.7%) (12). The phylogeny of the Han River isolates showed wide distribution, belonging to ST57 (*n* = 14), ST35 (*n* = 5), ST558 (*n* = 5), ST2 (*n* = 1), ST59 (*n* = 1), ST329 (*n* = 1), ST1094 (*n* = 1), and ST1099 (*n* = 1), while six strains could not be classified using the MLST scheme.

### ARG profiles of *S. epidermidis* genomes

**Fig 2:**
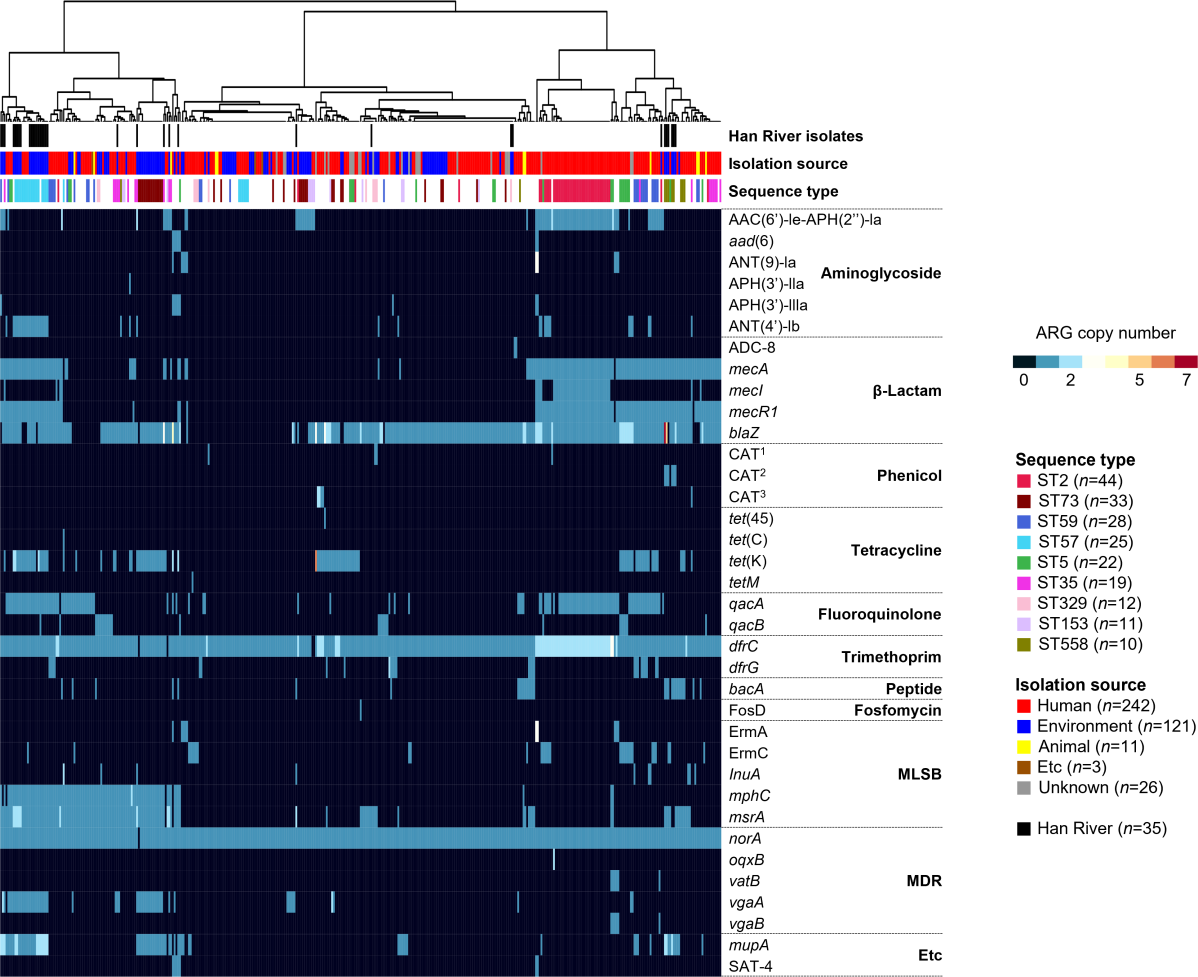
ARG profile of *Staphylococcus epidermidis* pangenome. The ARG profile-based clustering was performed using the Manhattan clustering method. Black color in the upper bar indicates the Han River isolates. Colors in the middle bar indicate the isolation sources of each strain. Etc indicates isolation sources other than humans, animals, and the environment. Colors in the lower bar indicate sequence types. CAT^1^, CAT^2^, and CAT^3^ indicate chloramphenicol acetyltransferase (CAT) from *Enterococcus faecalis*, *E. faecium*, and *S. intermedius*, respectively. The heatmap shows the copy number of ARGs.

The ARG profiles were determined for 405 *S*. *epidermidis* genomes with <50 contigs, and ARGs were detected in 403 genomes (Fig. 2). The genome-wide core ARGs, which were detected in more than 95% of *S. epidermidis* genomes, were identified to be the multidrug efflux pump-coding gene, *norA* (99% of 405 *S*. *epidermidis* genomes), and the trimethoprim resistance-coding gene, *dfrC* (98%) (Fig. 2). The genomes of *S. epidermidis* were found to harbor several different ARGs, including *blaZ* (76%), *mecA* (39%), *mecR1* (35%), and *mecI* (10%) genes conferring resistance to β-lactam antibiotics, and *msrA* (33%) and *mphC* (25%) genes conferring resistance to macrolide-lincosamide-streptogramin B (MLSB) antibiotics. The *S. epidermidis* genomes also contained the fluoroquinolone resistance gene, *qacA* (31%), tetracycline resistance gene, *tet*(K) (24%), aminoglycoside resistance gene, *aac(6′)-Ie-aph(2″)-Ia* (20%)*,* mupirocin resistance gene, *mupA* (18%), and the multidrug resistance gene, *vgaA* (14%).

**Fig 3:**
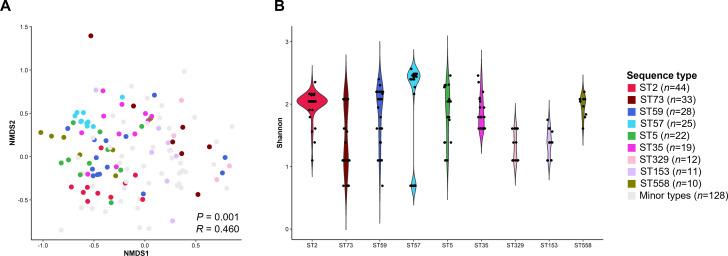
ARG diversity of *Staphylococcus epidermidis* associated with their sequence types. (**A**) Plot coordinates were determined by non-metric multidimensional scaling analysis of Bray–Curtis dissimilarity matrix, based on the ARG profiles. (**B**) The Shannon index was used to estimate the diversity of ARGs in each *S. epidermidis* genome. Minor types indicate the sum of each minor type (1 < *n* < 10).

To analyze the relationship between ARG profiles and sequence types (18), we selected genomes from sequence types with *n* ≥ 2 among 403 *S*. *epidermidis* genomes that contained any of the ARGs. Distance-based non-metric multidimensional scaling (NMDS) analysis revealed that resistome clusters were significantly separated according to sequence type [analysis of similarities (ANOSIM), *P* = 0.001, *R* = 0.460] (Fig. 3A). The number of ARGs encoded per genome was distinct among the sequence types (Fig. 3B). Among the major sequence types, hospital-associated ST2 (*n* = 44) showed the most conserved ARG profiles, whereas ST73, ST59, ST57, and ST5 exhibited relatively diverse ARG profiles across genomes (Fig. 3B). The MGE profiles were also distinct among sequence types (for plasmid-like sequences, ANOSIM, *P* = 0.001, *R* = 0.209; for transposases, ANOSIM, *P* = 0.001, *R* = 0.487) (Fig. S1). Integrase sequences were not identified in the 403 *S*. *epidermidis* genomes.

**Fig 4:**
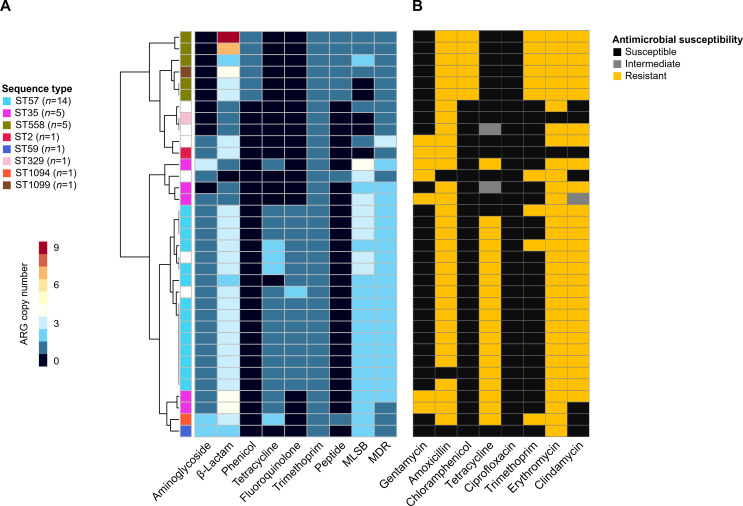
Genome-wide ARG profiles and resistance phenotypes of *Staphylococcus epidermidis* from the Han River isolates. (**A**) ARG profiles of *S. epidermidis* genomes from Han River isolates. The ARG profile-based clustering was performed using the Manhattan clustering method. Colors on the right side of the tree nodes indicate sequence types. The heatmap shows the copy number of the ARGs. (**B**) Antimicrobial susceptibility test results for the Han River isolates. Black, gray, and yellow colors indicate susceptible, intermediate, and resistant, respectively.

**Fig 5:**
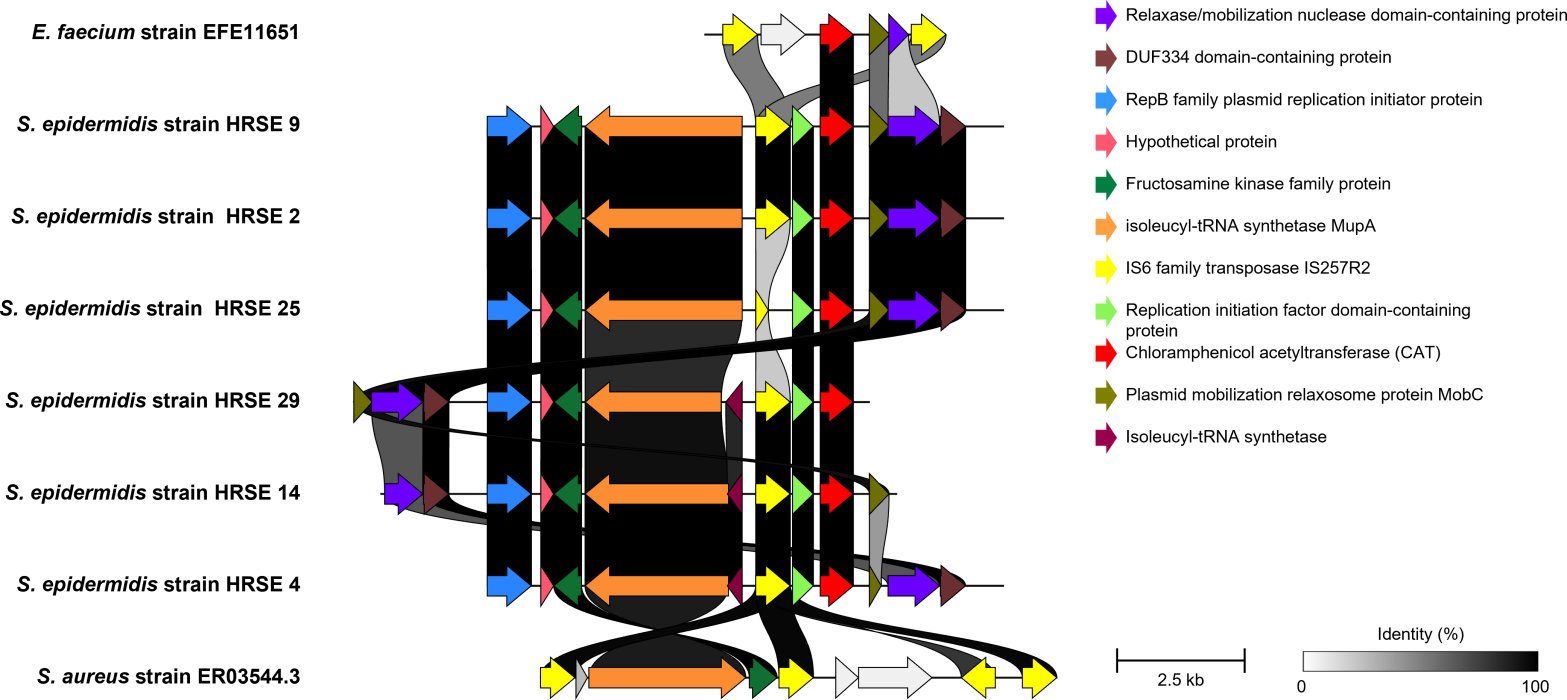
Plasmid-like sequences of the Han River isolates harboring chloramphenicol acetyltransferase genes (*cat*) and associated with IS6 family transposases. Pairwise alignment and visualization of gene clusters were performed using Clinker.

The ARG profiles of the Han River isolates were similar to those of the entire *S. epidermidis* genomes obtained from the public database with a notable prevalence of β-lactam and MLSB class ARGs in most of the genomes (Fig. 4). The presence of ARGs against β-lactams, phenicol, tetracycline, and MLSB was highly consistent with the resistance phenotypes of these isolates, whereas that of ARGs against aminoglycosides, fluoroquinolones, and trimethoprim was not (Fig. 4). Notably, the plasmid-like sequences of the Han River isolates belonging to ST558 (*n* = 5) and ST1099 (*n* = 1) harbored chloramphenicol acetyltransferase genes (*cat*) identical to those found in the plasmids of *Enterococcus faecium* (Fig. 5). These plasmid-like sequences were associated with insertion sequence 6 family transposases, homologous to those found in the plasmids of *S. aureus* (Fig. 5), which do not contain *cat* gene.

### Comparison of MLST, ARG, MGE, and VF profiles among *S. epidermidis* genomes of human, animal, and environmental isolates

**Fig 6:**
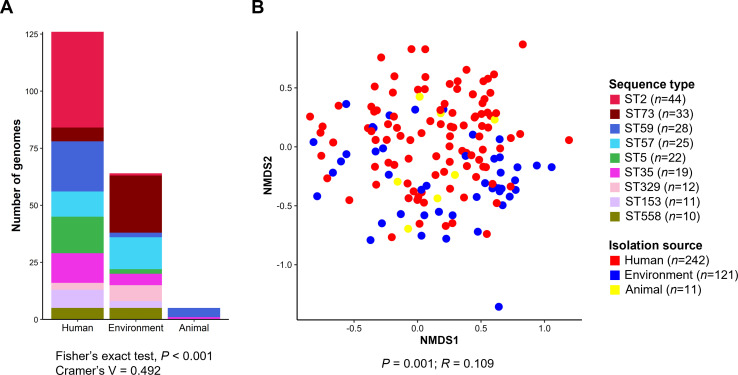
MLST distribution and ARG profiles of *Staphylococcus epidermidis* genomes among isolation sources. (**A**) MLST distribution among different isolation sources was analyzed using Fisher’s exact test and Cramer’s V. (**B**) Plot coordinates were determined by non-metric multidimensional scaling analysis of Bray–Curtis dissimilarity matrix, based on the ARG profiles.

MLST distribution was compared among human, animal, and environmental isolates of *S. epidermidis*. As a result, MLST composition significantly differed according to isolation sources (Fisher’s exact test, *P* < 0.001; Cramer’s V = 0.492) (Fig. 6A): ST2, ST59, and ST5 were significantly more abundant in *S. epidermidis* genomes of human origin than environmental genomes, whereas ST73 was highly dominant in environmental genomes. The ARG profiles of *S. epidermidis* genomes were clustered, but not clearly separated, among humans, animals, and the environment (ANOSIM, *P* = 0.001, *R* = 0.109) (Fig. 6B). Clusters of animals and environment were included within the human clusters, suggesting that most *S. epidermidis* strains were of human origin. Notably, ARG profiles of *S. epidermidis* genomes were distinguished among six different isolation countries (*n* ≥ 24) (ANOSIM, *P* = 0.001, *R* = 0.232) (Fig. S2). In the case of MGEs, the transposase profiles were clustered among different isolation sources (ANOSIM, *P* = 0.002, *R* = 0.055) (Fig. S3A), whereas the plasmid-like sequences were not significantly clustered (ANOSIM, *P* = 0.362, *R* = 0.001) (Fig. S3B). In addition, the VF profiles of *S. epidermidis* genomes were not associated with isolation sources (ANOSIM, *P* = 0.315, *R* = 0.009) (Fig. S3C).

### Comparison of the ARG and VF profiles between *S. epidermidis* and *S. aureus* genomes

**Fig 7:**
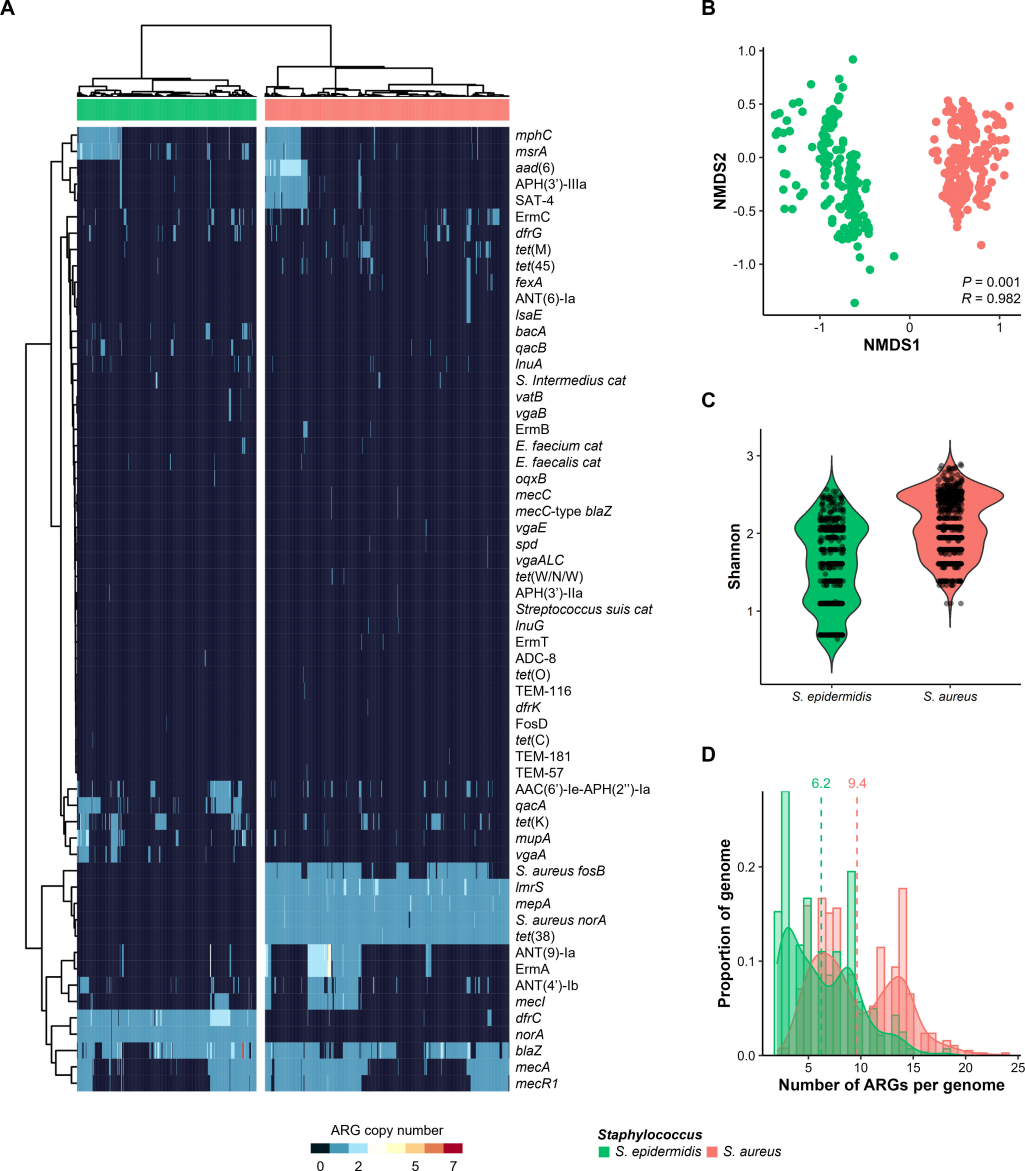
Comparison of ARG profiles between *Staphylococcus epidermidis* and *S. aureus* genomes. (**A**) The ARG profile-based clustering was performed using the Manhattan clustering method. The heatmap shows the copy numbers of ARGs. (**B**) The coordinates of the plot were determined by a non-metric multidimensional scaling analysis of Bray–Curtis dissimilarity matrix, based on ARG profiles. (**C**) The ARG diversity in each genome was estimated using the Shannon index. (**D**) Distribution of ARG copies per genome. The dotted lines indicate the average number of ARG copies in each species. Green and coral colors indicate *S. epidermidis* and *S. aureus*, respectively.

Phylogenetically adjacent *S. epidermidis* and *S. aureus* are both common commensals on human skin and are major causes of nosocomial infections. The overlapping ecological niches of these two species suggest that various inter-species interactions may occur. Therefore, we performed a comparative genomic analysis focusing on ARGs and VFs. Our results revealed that each species has a distinct ARG profile with certain genes found exclusively in each genome (Fig. 7A and B). *S. epidermidis* genomes displayed relatively more diverse ARG profiles than *S. aureus* genomes (Fig. 7C). *S. aureus* possessed a larger number of ARGs per genome (9.4 ARG copies) than *S. epidermidis* (6.2 ARG copies) (Fig. 7D). The genome-wide core ARGs in *S. epidermidis* were *dfrC* and *norA*, while *lmrS*, *S. aureus norA*, *mepA*, and *tet*(38) were the core ARGs in the *S. aureus* genomes; core ARGs were not shared between the two species (Fig. 7A). The *S. epidermidis* and *S. aureus* genomes encoded the β-lactam class ARGs, *blaZ* (76% and 75% of each genome, respectively), *mecA* (39% and 60%), *mecR1* (35% and 53%), and *mecI* (10% and 23%), and the MLSB class ARGs, *msrA* (33% and 16%) and *mphC* (25% and 15%) (Fig. 7A).

The VF profiles of *S. epidermidis* and *S. aureus* were more clearly distinguishable than their ARG profiles (Fig. S4A and B). The genome-wide core VFs of *S. epidermidis* included *tufa* (100% of *S. epidermidis* genomes)*, clpP* (99%)*, acpXL* (99%)*,* and *aur* (97%). In contrast, *S. aureus* consisted of 53 core VFs, including the four core VFs of *S. epidermidis* (Fig. S4A), indicating that the VF composition of *S. aureus* genomes was considerably more diverse (Fig. S4C) and abundant than that of *S. epidermidis* genomes. Notably, *esaG*, which is associated with the type VII secretion system, was the most abundant VF across all genomes (99% of *S. aureus* genomes) (Fig. S4A). *S. aureus* possessed an enormously larger number of VFs per genome (80.8 VF copies) than *S. epidermidis* (4.8 VF copies) (Fig. S4D).

## DISCUSSION

The increasing prevalence of multidrug-resistant *S. epidermidis* has become a serious problem constraining treatment options in clinical settings. It has a relatively small genome of approximately 2.5 Mb, and 20% of its genome is composed of variable structures (19), which suggests that this opportunistic pathogen has an open pan-genome and high potential to acquire new genetic traits via MGEs (19). Furthermore, evidence of horizontal gene transfer between *S. epidermidis* genomes from different skin sites involving antibiotic resistance and virulence genes has been reported (20). The flexible genome structures of *S. epidermidis* and horizontal gene transfer contribute significantly to the unpredictability of the *S. epidermidis* genome (7).

In this study, we highlighted the ARGs present in *S. epidermidis* genomes by analyzing comprehensive and high-quality genomes obtained from diverse habitats. Previous genomic studies have focused only on the clinical isolates, which may lead to biased views and hinder our understanding of the genetic structures of antibiotic resistance across different habitats. Therefore, a comparison of the entire *S. epidermidis* genomes from the One-Health perspective is critical. Although *S. epidermidis* is primarily found on human skin, 33% of the genomes analyzed in our study was obtained from various habitats other than human skin, including the environment (30%) and animals (3%). In the present study, we isolated 35 environmental strains of *S. epidermidis* from the Han River and those genomes were analyzed. The ARG profiles of these isolates were similar to those of the entire *S. epidermidis* genomes obtained from the public database. The genome-predicted ARG profiles were not perfectly consistent with experimentally determined resistance phenotype (Fig. 4). This inconsistency may be due to several reasons: ARGs may not be expressed under certain conditions. ARGs may be mutated and become non-functional. The presence of multidrug efflux pumps can also make resistance phenotype to certain antibiotics unpredictable. ARGs not yet discovered may confer resistance phenotype. Interestingly, genomes of the six environmental isolates from the Han River sequenced in this study contained plasmid-like sequences harboring *cat* genes identical to those of *E. faecium*. These ARGs have never been identified in *S. epidermidis* genomes. Furthermore, these sequences were associated with IS6 family transposases, which are homologous to those of *S. aureus*. These results suggest that ARGs structured in the transposon of plasmid may have been transmitted between these Gram-positive pathogens (21, 22).

Previous studies have suggested that the genotype of *S. epidermidis* is associated with infection and host disease (12, 23), and its ARG profile may also be closely related to its sequence type (12, 24). Our results revealed that *S. epidermidis* ARG profiles based on their genomes could be distinguished according to different sequence types. Hospital-associated ST2 was found to have relatively conserved ARG profiles, whereas sequence types isolated from various habitats, such as ST73, ST59, ST57, and ST5, possessed more diverse ARG profiles, suggesting that ARG diversity within a specific sequence type may be influenced by ecological niches.

Our study revealed that the ARG and VF profiles of *S. epidermidis* and *S. aureus* could be clearly distinguished at the genome level. Despite overlapping ecological niches, gene transfer between staphylococcal species is believed to be rare owing to the presence of restriction modification systems and clustered regularly interspaced short palindromic repeats (CRISPR) loci that degrade foreign DNAs (25, 26). However, several studies have indicated that the proportion of CRISPR loci within staphylococci is lower than that reported previously (27). In addition, multiple lines of evidence regarding the inter-species transfer of genes related to antibiotic resistance, pathogenicity, and metal resistance between *S. epidermidis* and *S. aureus* have been documented (22, 28, 29). The transfer of genes may herald the emergence of *S. aureus* lineages with increased antibiotic resistance and virulence, as the genomic flexibility of *S. epidermidis* renders it likely to act as a genetic reservoir for pathogens such as *S. aureus* (20, 30).

In conclusion, our results underscore that the genome-wide ARG profiles of *S. epidermidis* are well characterized among their sequence types. Our study contributes to a more comprehensive understanding of *S. epidermidis* antibiotic resistance at the pangenome level and provides a genomic basis for variations in resistance and virulence between staphylococcal pathogens *S. epidermidis* and *S. aureus*. Addressing how and why these two species from a similar ecological niche have evolved differently to possess distinct antibiotic resistance and virulence genes are the next steps for elucidating genomic demarcation.

## MATERIALS AND METHODS

### Isolation and identification of *S. epidermidis*

Twenty-two *S. epidermidis* strains were isolated from the Han River, South Korea, from 2016 to 2019 using Mueller–Hinton agar (Difco, Detroit, MI, USA) supplemented with various antibiotics. The antibiotics used were amoxicillin (16 mg/L), chloramphenicol (16 mg/L), erythromycin (2 mg/L), gentamicin (8 mg/L), lincomycin (2 mg/L), sulfamethoxazole (40 mg/L), and tetracycline (4 mg/L). All antibiotics were obtained from Sigma-Aldrich (St. Louis, MO, USA). Thirteen strains were isolated from the Han River in May 2019 using the Baird–Parker medium supplemented with egg yolk (Difco). These isolates were identified using the EzBioCloud database (https://www.ezbiocloud.net/resources/16s_download) (31) based on their 16S rRNA gene sequences.

### Antimicrobial susceptibility test

Antimicrobial susceptibility test was performed using the disk diffusion assay according to the EUCAST guidelines, 2016. The amount of antibiotic in each disk was as follows: amoxicillin (10 µg), cephalexin (30 µg), chloramphenicol (30 µg), ciprofloxacin (5 µg), clindamycin (2 µg), colistin (10 µg), erythromycin (15 µg), fosfomycin (200 µg), gentamicin (10 µg), linezolid (10 µg), meropenem (10 µg), streptomycin (10 µg), sulfamethoxazole (50 µg), rifampicin (5 µg), tetracycline (30 µg), trimethoprim (5 µg), tylosin (30 µg), and vancomycin (30 µg). All disks were obtained from Liofilchem (Roseto, Italy).

### Whole-genome sequencing and assembly

A total of 35 environmental isolates from the Han River were subjected to whole-genome sequencing. Genomic DNA was extracted using the DNeasy blood and tissue kit (Qiagen, Hilden, Germany) and prepared using the ligation sequencing kit (SQK-LSK109; Oxford Nanopore Technologies, Oxford, UK), following the manufacturer’s protocols. Whole-genome sequencing was performed using Oxford Nanopore MinION flow cells (R9.4.1 FLO-MIN106; Oxford Nanopore Technologies) at DNA Link (Seoul, Korea). Basecalling on the raw signal data was performed using the Guppy v6.0.7. The draft genomes were *de novo* assembled using Flye, Miniasm, and Raven implemented in Trycycler v0.5.3 (32).

### Phylogenomic analysis

The genome assembly data of 967 *S*. *epidermidis* strains and *S. aureus* DSM 20231 (an outgroup) obtained from the NCBI RefSeq database, and 35 *S*. *epidermidis* genomes from the environmental isolates sequenced in this study, were used for phylogenomic analysis. A phylogenomic tree was constructed using the core genes shared among all compared genomes. To identify the core genes, a bidirectional best BLASTP search was performed for protein-coding sequences across the entire genome. A gene was considered to be a core gene when the best hits for that gene were found in more than 95% of the compared genomes. The nucleotide sequences of each core gene were aligned using MAFFT v7 (33), and the resulting alignments were concatenated. Maximum-likelihood phylogenetic trees were constructed from the concatenated genes by running FastTree2 as a general-time reversible model of nucleotide substitutions (34). Interactive Tree of Life v5 was used to visualize the phylogenomic tree (35). MLST was performed using the MLST software (36) based on the PubMLST database. Accurate taxonomic identification was performed using ANI values obtained using the FastANI tool (37).

### Analysis of ARGs, VFs, and MGEs in the genome sequences

For the analysis of ARGs, VFs, and MGEs, 405 genome sequences (Data Set S1) were used, including 370 *S*. *epidermidis* genomes with <50 contigs selected from the NCBI RefSeq database (Data Set S2) and 35 river genomes sequenced in this study. For *S. aureus*, 550 genomes were randomly selected. ARGs were detected based on the Comprehensive Antibiotic Resistance Database using the Resistance Gene Identifier software with strict cutoff values (38). Shannon diversity indices of the ARG and VF profiles were estimated using the vegan R package (39). Clinker was used to visualize gene clusters (40). VFs were annotated using a BLASTP search with the core data set of the virulence factor database with a cutoff of ≥70% identity and ≥70% query coverage. The ARG and VF profiles were clustered using a segmentation algorithm that clustered points based on the Manhattan distance in the pheatmap R package (41). A heatmap was constructed using the pheatmap R package (41). Transposases were annotated using a BLASTP search and the IS database with a cutoff of ≥80% identity and ≥80% query coverage. Plasmid-like sequences were identified using a BLASTN search against plasmid sequences downloaded from the NCBI RefSeq database with ≥80% identity and ≥2,000 bp alignment length. Differences in ARG, VF, and MGE profiles among each sequence type, isolation source, isolation country, or species were analyzed using NMDS, and significance was tested using the ANOSIM in the vegan R package (39). MLST distribution among different isolation sources was analyzed using Fisher’s exact test in R and Cramer’s V in the rcompanion R package (42, 43).

## Data Availability

Raw nucleotide sequence reads and assembled genome sequences of the Han River isolates were deposited in the NCBI BioProject under the accession number PRJNA998543.

## SUPPLEMENTAL MATERIAL

Data Set S1 msystems.00226-24-s0001.xlsx *Staphylococcus epidermidis* genome assemblies used for ARG, VF, and MGE analysis.

Data Set S2 msystems.00226-24-s0002.xlsx Detailed information of 370 genomes downloaded from RefSeq.

Supplemental figures and table msystems.00226-24-s0003.pdf Fig. S1 to S4, Table S1.

ASM does not own the copyrights to Supplemental Material that may be linked to, or accessed through, an article. The authors have granted ASM a non-exclusive, world-wide license to publish the Supplemental Material files. Please contact the corresponding author directly for reuse.
