# Supplementary material for: Comprehensive genomic landscape of antibiotic resistance in Staphylococcus epidermidis
Source: mSystems. 2024 May 10;9(6):e00226-24. doi: 10.1128/msystems.00226-24 (PMC11237394; doi:10.1128/msystems.00226-24)
Supplement: Supplemental figures and table — Fig. S1 to S4, Table S1. [file msystems.00226-24-s0003.pdf]

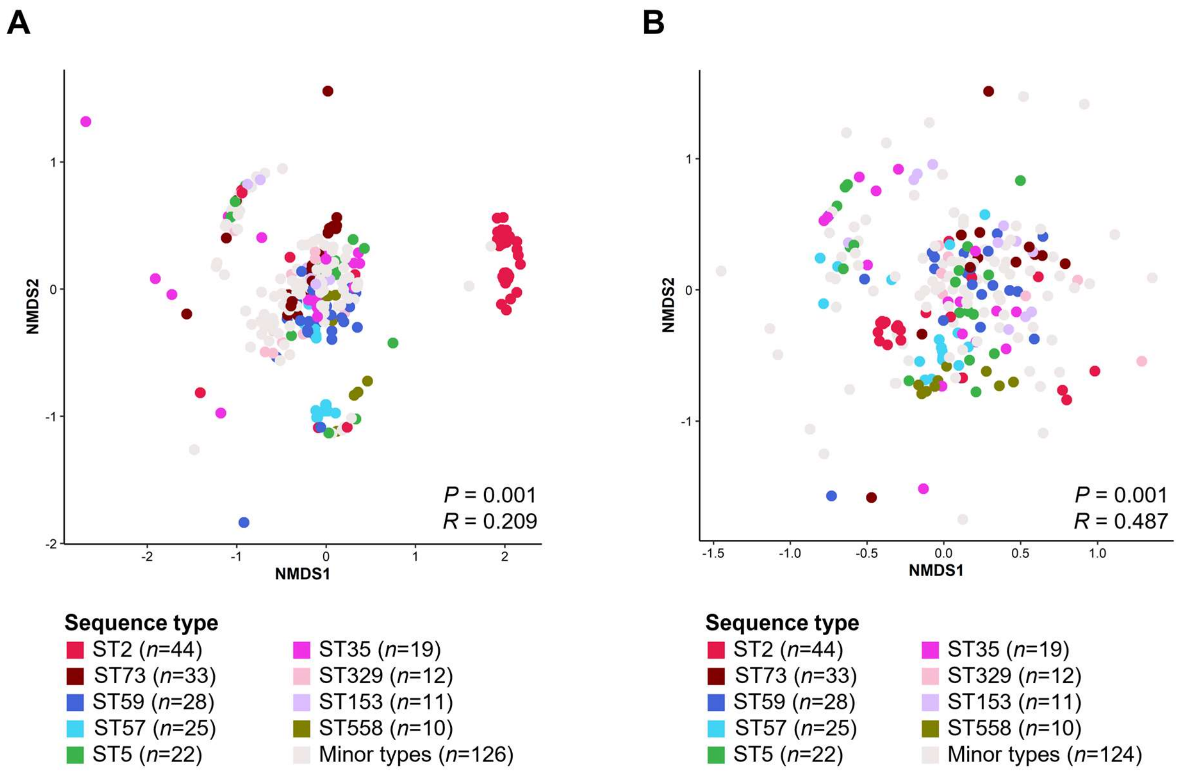

FIG S1 Diversity of mobile genetic elements of *Staphylococcus epidermidis* genomes associated with their sequence types. Plot coordinates were determined using non-metric multidimensional scaling analysis of Bray–Curtis dissimilarity matrix, based on the assigned name of plasmid-like sequences (**A**) and transposase profiles (**B**). Minor types indicate the sum of each minor type (1 < n < 10).

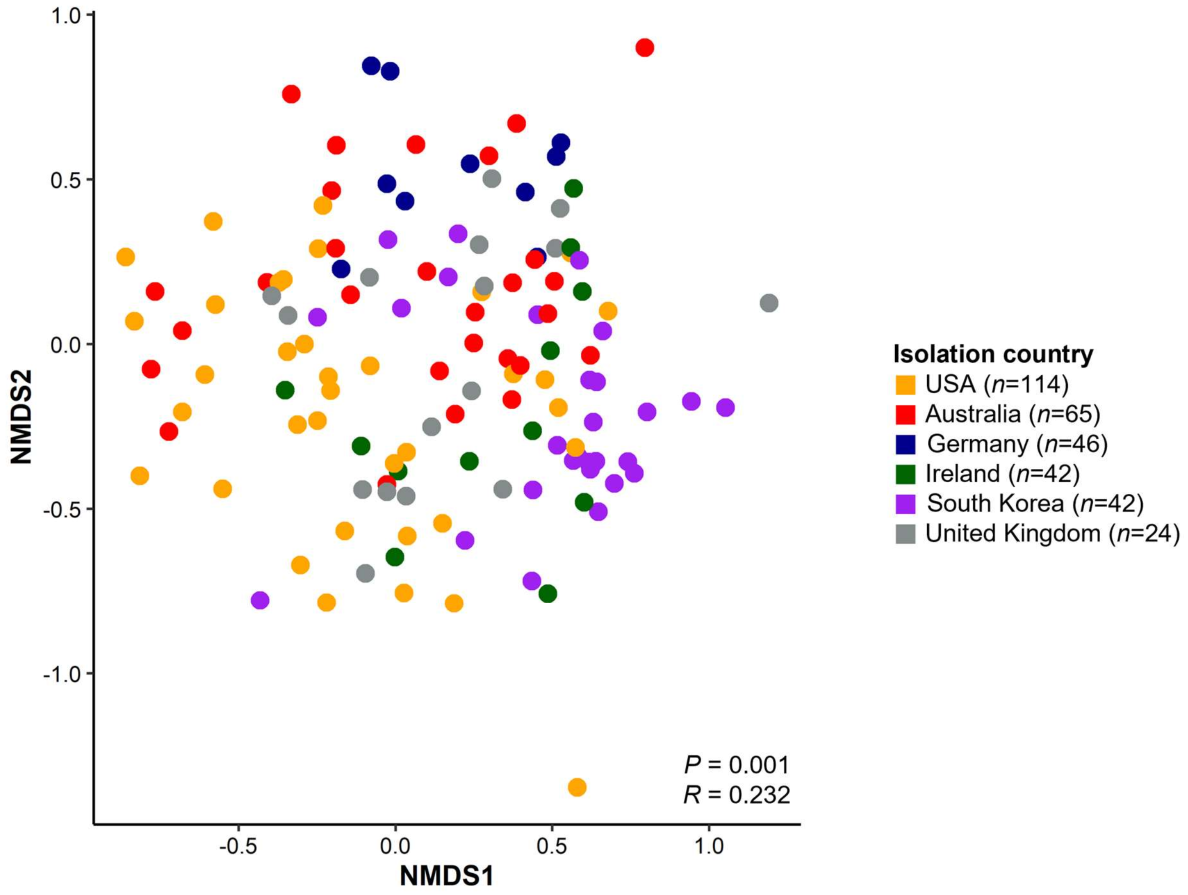

FIG S2 ARG diversity of *Staphylococcus epidermidis* genomes associated with their isolation countries. Plot coordinates were determined using non-metric multidimensional scaling analysis of Bray–Curtis dissimilarity matrix, based on the ARG profiles.

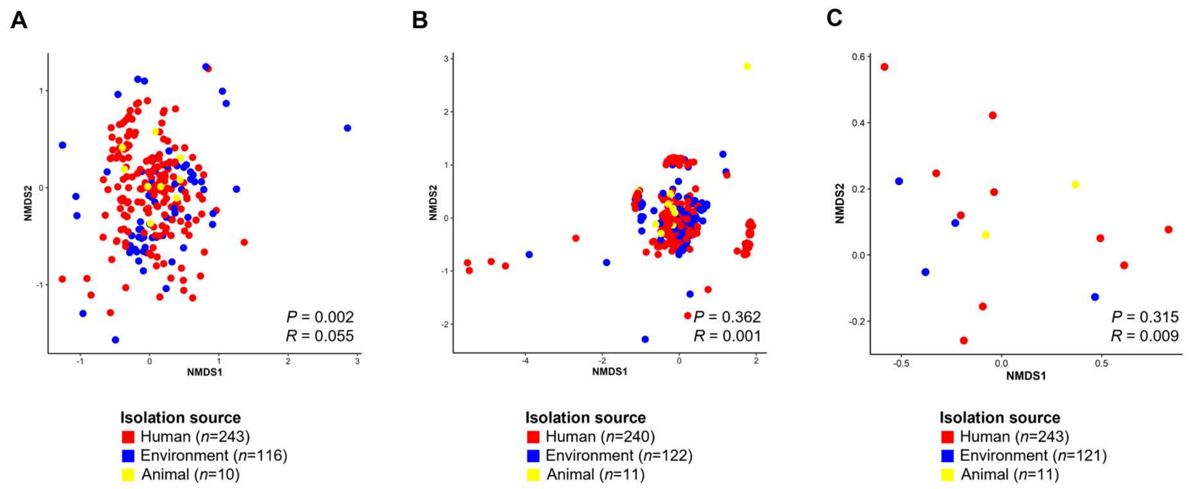

FIG S3 Diversity of mobile genetic elements and virulence factors (VFs) of *Staphylococcus epidermidis* genomes associated with their isolation sources. Plot coordinates were determined using non-metric multidimensional scaling analysis of Bray–Curtis dissimilarity matrix, based on the transposase profiles (**A**), assigned name of plasmid-like sequences (**B**), and VF profiles (**C**).

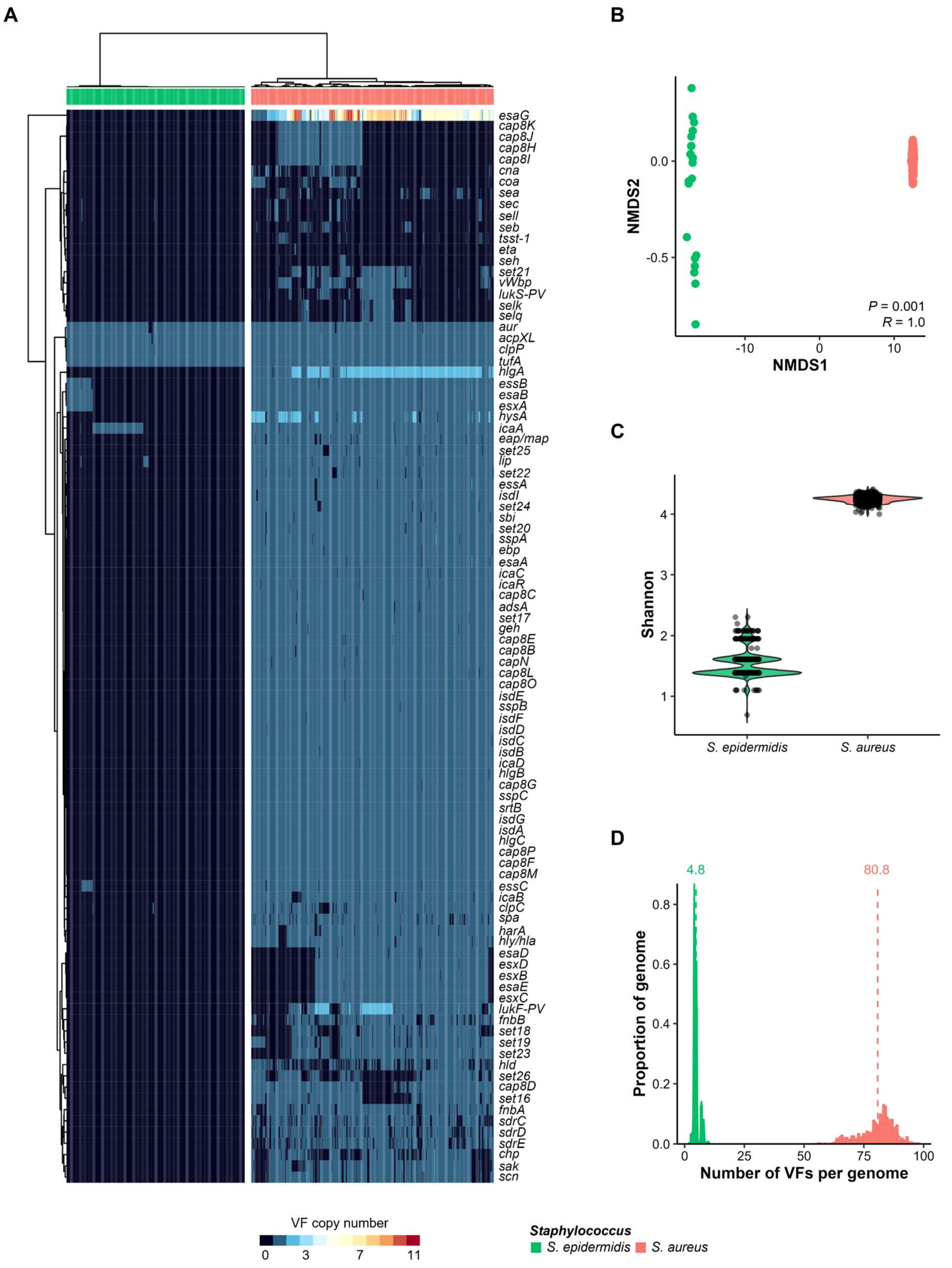

FIG S4 Comparison of virulence factor (VF) profiles between *Staphylococcus epidermidis* and *S. aureus* genomes. (**A**) The VF profile-based clustering was performed using the Manhattan clustering method. The heatmap shows the copy number of VFs per genome. (**B**) The

coordinates of the plots were determined using non-metric multidimensional scaling analysis of Bray–Curtis dissimilarity matrix based on the VF profiles. (**C**) The diversity of VFs in each genome was estimated using the Shannon index. (**D**) Distribution of VF copies per genome. Dotted lines indicate the average number of VF copies in each species. Green and coral colors indicate *S. epidermidis* and *S. aureus*, respectively.

TABLE S1 Genome summary of *Staphylococcus epidermidis* isolates sequenced in this study.

Strain	Genome size (bp)	G + C Content (%)	No. of contigs	No. of total genes	No. of protein coding genes	No. of antibiotic resistance genes	No. of rRNA genes	No. of tRNA genes
HRSE 1	2,411,565	32.3	4	2,409	2,330	4	19	59
HRSE 2	2,551,672	32.0	3	2,562	2,481	10	19	61
HRSE 3	2,456,435	32.2	5	2,470	2,390	12	19	60
HRSE 4	2,539,921	32.1	2	2,540	2,459	9	19	61
HRSE 5	2,459,725	32.2	6	2,469	2,389	12	19	60
HRSE 6	2,470,176	32.2	4	2,472	2,392	13	19	60
HRSE 7	2,313,048	32.0	5	2,364	2,285	11	19	59
HRSE 8	2,509,443	32.2	6	2,622	2,542	13	19	60
HRSE 9	2,571,553	32.1	6	2,656	2,575	9	19	61
HRSE 10	2,575,973	32.3	3	2,630	2,549	7	19	61
HRSE 11	2,450,675	32.2	4	2,459	2,379	14	19	60
HRSE 12	2,450,674	32.2	4	2,475	2,395	13	19	60
HRSE 13	2,450,641	32.2	4	2,460	2,380	15	19	60
HRSE 14	2,619,279	31.9	4	2,663	2,582	16	19	61
HRSE 16	2,621,594	32.1	6	2,698	2,617	14	19	61
HRSE 17	2,475,053	32.2	5	2,535	2,455	12	19	60
HRSE 18	2,481,308	32.2	6	2,524	2,444	13	19	60
HRSE 19	2,434,987	32.2	4	2,489	2,409	11	19	60
HRSE 20	2,359,389	32.2	6	2,536	2,456	13	19	60
HRSE 21	2,564,730	32.0	4	2,644	2,565	3	19	59
HRSE 22	2,450,983	32.2	5	2,512	2,432	13	19	60
HRSE 23	2,513,899	32.1	5	2,546	2,466	13	19	60
HRSE 24	2,408,822	32.2	5	2,471	2,392	13	19	59
HRSE 25	2,539,585	32.1	2	2,616	2,535	8	19	61
HRSE 26	2,447,742	32.2	4	2,495	2,415	12	19	60
HRSE 27	2,506,526	32.2	3	2,620	2,540	10	19	60
HRSE 28	2,469,566	32.2	4	2,517	2,437	14	19	60
HRSE 29	2,613,681	31.9	4	2,700	2,619	14	19	61
HRSE 30	2,456,668	32.2	3	2,482	2,402	6	19	60
HRSE 31	2,431,259	32.2	4	2,868	2,788	9	19	60
HRSE 32	2,456,176	32.2	5	2,526	2,446	14	19	60
HRSE 33	2,457,474	32.2	2	2,506	2,426	3	19	60
HRSE 34	2,572,932	32.0	5	2,629	2,549	11	19	60
HRSE 35	2,537,170	32.1	3	2,641	2,565	6	19	59
HRSE 36	2,469,905	32.2	5	2,594	2,514	14	19	60
